# Shift work disrupts Immune regulation of the transcriptome in hospital nurses

**DOI:** 10.3389/fpubh.2026.1859265

**Published:** 2026-09-22

**Authors:** Huan-Yan Hu, Xiang-Feng Xu, Ze Yu, Li Fang, Ke-Xin Fang

**Affiliations:** 1 Department of Rheumatology, Zhoushan Hospital, Wenzhou Medical University, Zhoushan, Zhejiang, China; 2 Department of Nursing, Zhoushan Hospital, Wenzhou Medical University, Zhoushan, Zhejiang, China; 3 Laboratory of Cytobiology and Molecular Biology, Zhoushan Hospital, Wenzhou Medical University, Zhoushan, Zhejiang, China

**Keywords:** circadian rhythm disruption, immune dysregulation, jak1, shift work, TRIM22

## Abstract

**Introduction:**

Shift work, especially night shifts in medicine, disrupts nurses‘ circadian rhythms, potentially causing immune dysregulation and health risks, with significant economic and medical burdens. The molecular mechanisms of shift work-related immune disorders are understudied. This research analyzes transcriptomes of nurses' peripheral blood mononuclear cells (PBMCs) from different shifts to identify key differentially expressed genes and explore their roles in immune regulation.

**Method:**

This study combined bioinformatics and animal experiments, analyzing the GSE122541 dataset for DEGs in day and night shift nurses' PBMCs. WGCNA identified core gene modules linked to night shift. Protein interaction networks were analyzed for hub genes, with expression validated in a circadian rhythm disorder mouse model.

**Results:**

The analysis found many differentially expressed genes in night shift nurses' PBMCs, mainly in immune pathways like T-cell receptor signaling and differentiation. WGCNA identified gene modules linked to night shifts, intersecting to reveal 8 candidate hub genes, with TRIM22 and JAK1 core in the network and downregulated in nurses. Mouse models showed Trim22 and Jak1 also decreased during nighttime activity, indicating their downregulation is a common trait in circadian rhythm disorders, involving interferon-γ and NF-κB immune signaling.

**Conclusion:**

This study shows that night shift work disrupts circadian rhythm and downregulates immune genes like *TRIM22* and *JAK1* in PBMCs. These genes may link shift work to immune imbalance, are potentially associated with IFN-γ and NF-κB pathways, offering potential targets for understanding shift work's health risks and developing interventions.

## 1 Introduction

Shift work, particularly in healthcare settings, has emerged as a significant concern due to its potential adverse effects on the immune system and overall health of workers (1). Hospital nurses, who often engage in night shifts, are particularly vulnerable to disruptions in circadian rhythms, which can lead to various health complications, including immune dysregulation (2, 3). The economic burden of such health issues is substantial, as they not only affect the wellbeing of the nurses but also compromise patient care and increase healthcare costs. Current diagnostic and treatment strategies for immune-related disorders associated with shift work are limited, primarily focusing on symptomatic relief rather than addressing the underlying molecular mechanisms (4, 5). Furthermore, the existing studies have not fully explored the specific gene expression changes in the peripheral blood mononuclear cells (PBMCs) of nurses from different shifts, nor have they clarified the complex relationship between these genes and the circadian rhythm. This highlights the necessity of our current study, which aims to determine the differentially expressed genes (DEGs) in the PBMCs of day shift and night shift nurses, analyze the co-expression modules, and explore the role of key genes in immune regulation.

Day-night shift work, is a well-established occupational exposure associated with circadian disruption and adverse health outcomes, including metabolic disorders, cardiovascular disease, and an increased risk of certain cancers (6–11). A growing body of epidemiological and clinical evidence suggests that this health burden may be mediated, in part, by dysregulation of immune system. Previous studies have linked circadian misalignment to altered inflammatory cytokine profiles and impaired immune cell function (12). However, the current research on the molecular mechanisms and key transcriptional regulatory factors underlying immune dysfunction associated with shift work is still not comprehensive enough.

Our work directly addresses this by integrating bioinformatic analysis of human transcriptomic data from shift-working nurses with experimental validation in a murine model. We focus on the exposure of consecutive night-shift work and its impact on the PBMC transcriptome. Leveraging GSE122541 dataset, we identified differentially expressed genes (DEGs) and, through Weighted Gene Co-expression Network Analysis (WGCNA) (13), pinpointed core gene modules correlated with night-shift status. Intersection of these analyses revealed 8 candidate hub genes. Subsequent protein-protein interaction network analysis prioritized TRIM22 and JAK1 as central regulators.

Our study aimed to combine bioinformatics with animal experiments to jointly confirm that *TRIM22* and *JAK1* are the potential hub factors, and their downregulation in peripheral blood mononuclear cells is a common feature of circadian rhythm disruption caused by night shifts. These findings indicated specific interferon-γ and NF-κB-related immune signaling pathways in this process.

## 2 Materials and methods

### 2.1 Data sources and gene expression profiles

We scoured the GEO database for high-throughput functional genomics research related to circadian rhythm of nurses. The raw data derived from the GSE122541 (14). Statistical analysis, error identification, data refinement, and organization were conducted using the limma package, thereby enhancing data management capabilities. For data normalization, the Robust Multi-array Average method was employed. A comprehensive analysis was performed by examining the gene expression datasets.

### 2.2 Statistical analysis

GraphPad Prism 8.0 was used for the data processing and statistical analysis. The results of experimental data from six independent replicates are presented as mean ± SD. Student's *t* test was used for comparisons between two independent sample groups. *P* value < 0.05 was considered to indicate statistical significance.

### 2.3 Supplementary method

Supplementary files provide the details on the methods for identifying DEGs, conducting functional enrichment analysis, performing Gene-MANIA analysis, WGCNA analysis and module identification, Day-night rhythm experiment, RNA extraction, reverse transcription and RT-qPCR.

## 3 Results

### 3.1 Identification of DEGs in GSE122541 dataset

The fowchart of our research design is shown in Supplementary Figure 1. Firstly, samples from dataset GSE122541 were initially categorized into two groups: the hospital nurses working night-shift (*n* = *9*) and day-shift (*n* = *8*), followed by an analysis of the differentially expressed genes (DEGs) within these cohorts. Subsequently, we identified the DEGs of GSE122541 dataset (Figures 1A, B). A thorough GO analysis was conducted on the DEGs derived from GSE122541 (Figure 1C and Supplementary Figures 2A, B). These results indicated these genes that were upregulated in night-shift nurses were mainly associated with the T cell receptor signaling, T cell differentiation, MHC complex assembly and immune response-activating cell surface (Figure 1C). Based on these findings, we show that the shift-work pattern can affect the expression of immune regulation-related genes in PBMCs of these nurses.

**Figure 1:**
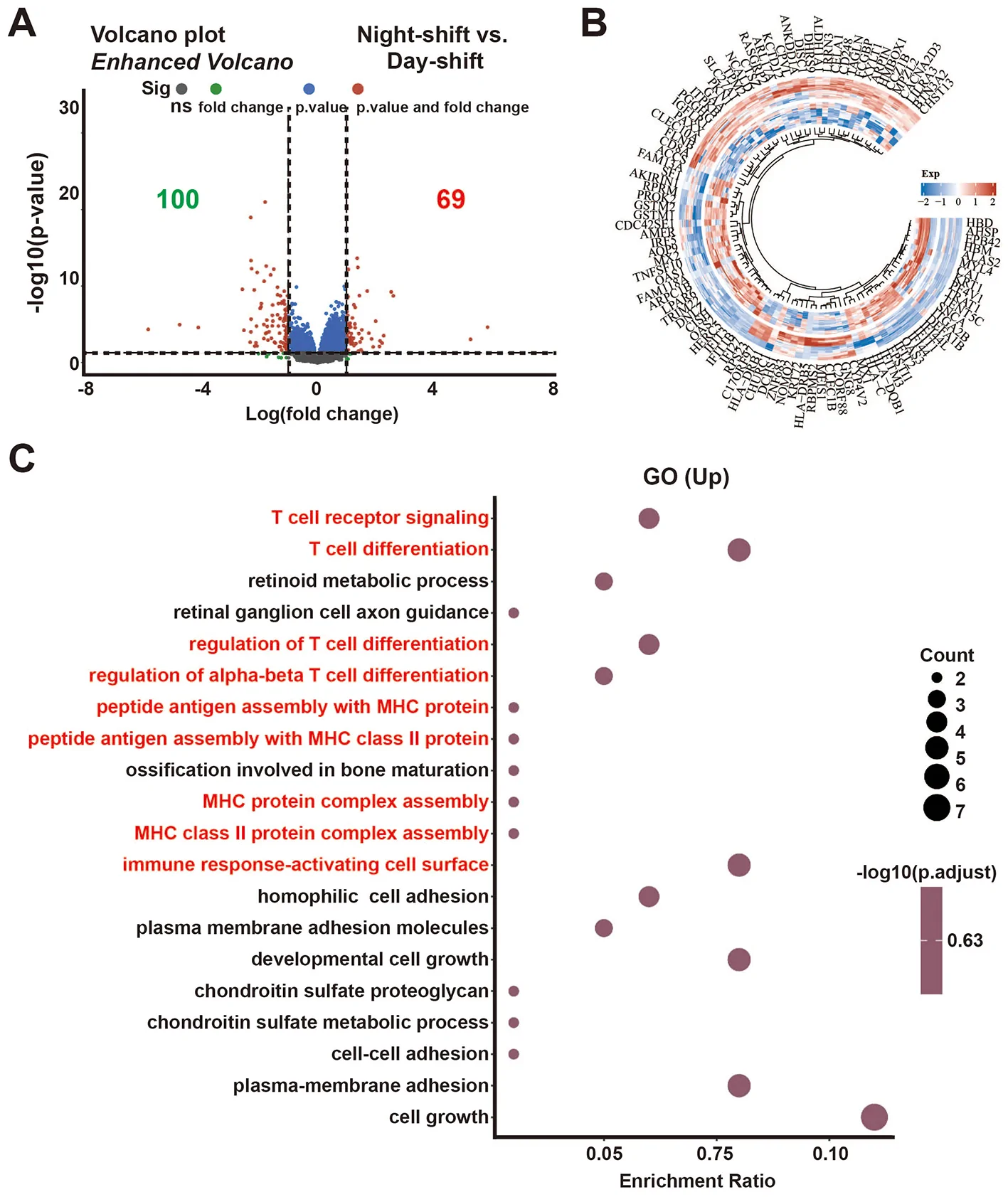
Analysis of differentially genes and GO in the GSE122541 dataset. **(A, B)** Volcano and heat-map of differential genes in GSE122541; **(C)** GO term enrichment results of the upregulated differential genes.

### 3.2 Identification of co-expression gene modules and hub genes in the DEGs

We employed WGCNA to discern co-expression gene modules within the dataset of day-night shift nurses, GSE122541. With the soft threshold power set at 6, a scale independence of 0.86 was achieved (Figure 2A). The samples from the GSE122541 were categorized into the day-shift nurse group and night-shift nurse group, with no outliers identified (Figure 2B). Hierarchical clustering and dynamic branch cutting methods were applied to gene dendrogram, when the cut height was established at 0.25 and minimum module size was set to 30, 11 distinct co-expression modules were derived through the dynamic tree cutting (Figures 2C, D).

**Figure 2:**
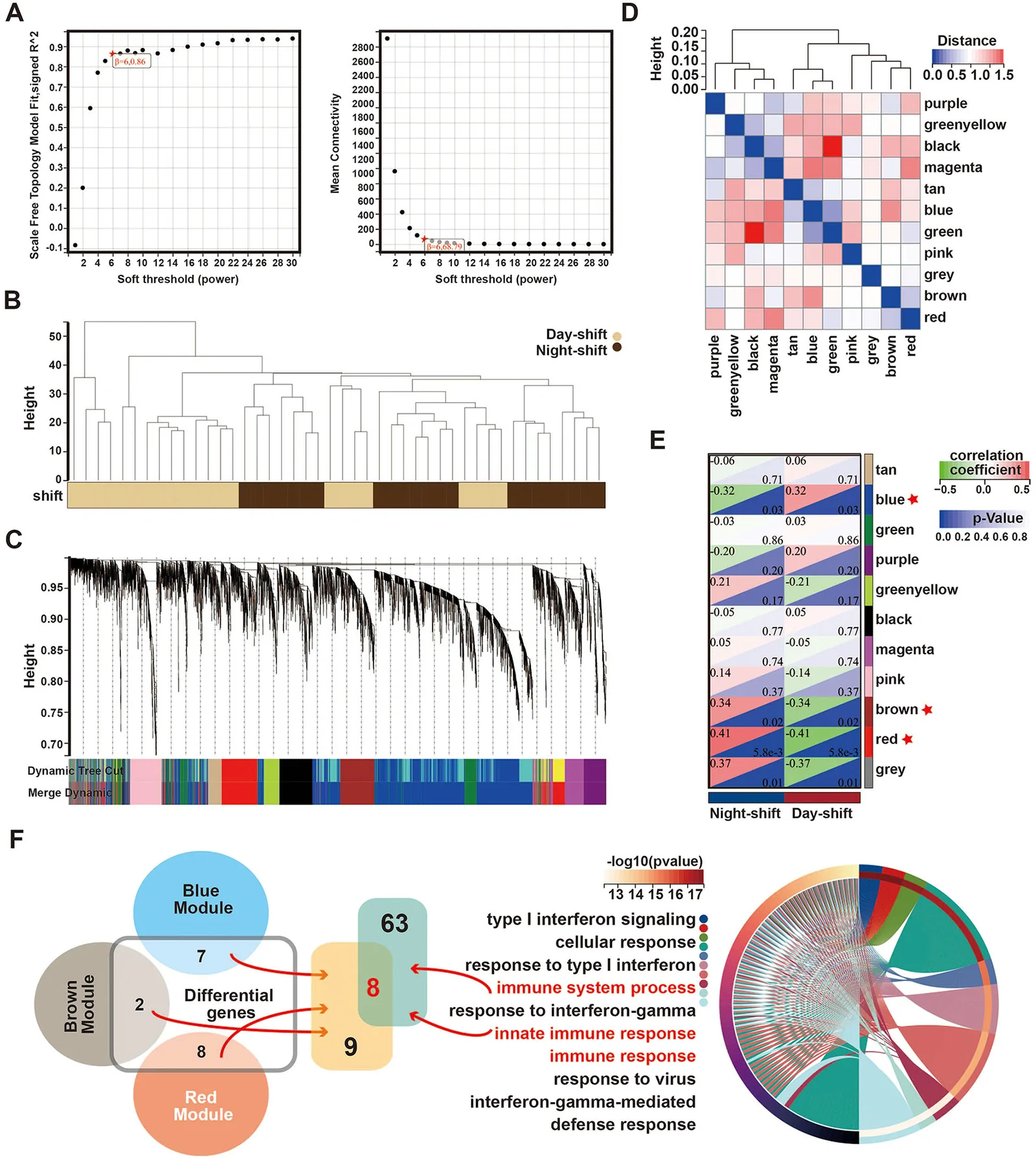
WGCNA analysis of the day-night shift nurses via GSE122541. **(A)** Analysis of network topology for various soft thresholds (β); **(B)** Clustering dendrogram of all samples; **(C)** The gene dendrograms obtained by average linkage hierarchical clustering; **(D)** Topological Overlap Matrix (TOM) of genes selected for the weighted co-expression network analysis depicts in Heatmap; **(E)** Module-trait associations; **(F)** Venn diagram of key module genes vs. DEGs related to the immune system.

Subsequently, correlation analyses of each module with the shift-work pattern were conducted. The brown and red modules exhibited stronger positive correlation with night-shift in nurses, while the blue module demonstrated the most significant negative correlation with night-shift of nurses (Figure 2E). Furthermore, correlation analysis between MM and GS indicated that these genes were highly correlated with both modules we selected (Supplementary Figure 3).

At the same time, based on the DEGs involved in immune regulation that obtained previously between the day-night shift nurses, the Venn analysis revealed that there were eight DEGs present in the three core modules, including *ROGDI, GAA, PKN1, TRIM22, KRT1, LAX1, ARPC1B* and *JAK1* (Figure 2F).

### 3.3 Reversed light-dark schedule experiment confirmed that *TRIM22* and *JAK1* are the potential hub factors

First, we analyzed the differences of these eight hub genes among nurses. Results showed *ROGDI, GAA* and *PKN1* were significantly elevated in the PBMCs of night shift nurses, while the other five genes (*TRIM22, KRT1, LAX1, ARPC1B* and *JAK1*) were decreased (Figure 3A). Then, the Gene- MANIA analysis revealed that *TRIM22* and *JAK1* had the most interaction among the eight genes involved in a variety of cell signals, including response to interferon-γ and I-κB kinase/NF-κB (Figures 3B, C). Besides, *TRIM22* and *JAK1* also were downregulated in the night-shift nurses. These findings indicated *TRIM22* and *JAK1* were significantly downregulated among the PBMCs of nurses with three consecutive night shifts (Figure 3D). This further suggested that the immune regulatory functions or signals mediated by TRIM22 and JAK1 may be inhibited or absent.

**Figure 3:**
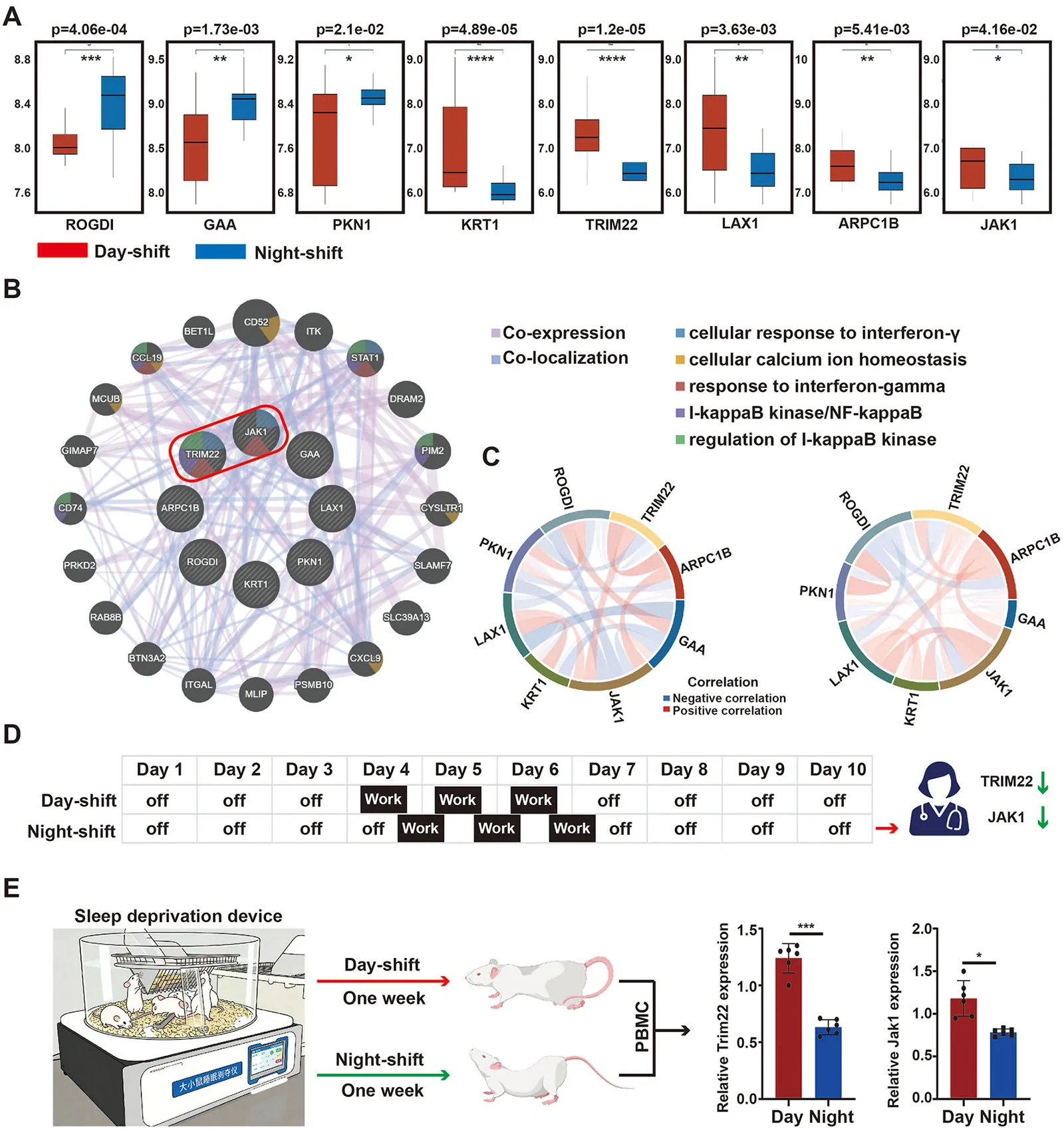
The circadian rhythm experiment determined the core factor. **(A)** Differential expression analysis of 8 hub genes in PBMCs samples of night-shift nurses; **(B)** Gene-MANIA was used to analyze the function and correlation of 8 hub genes; **(C)** Correlation expression analysis of these genes; **(D)** Shift work schedule of nurses enrolled in the GSE122541; **(E)** Using sleep deprivation devices to conduct experiments on the circadian rhythm of mice and the detection and verification of genes *TRIM22* and *JAK1* in the PBMCs of mouse.

Finally, the reversed light-dark schedule experiment on mouse further confirmed the previous conclusion, that is, compared to normal mice that are active during the day, the levels of *Trim22* and *Jak1* in PBMCs of mouse which were active at night for a consecutive week were significantly decreased (Figure 3E). Therefore, we believe *TRIM22* and *JAK1* are the **potential hub factors** responsible for the imbalance of the immune system in night shift nurses.

## 4 Discussion

Shift work, particularly in healthcare settings, poses significant challenges to the immune health of workers, especially hospital nurses who frequently engage in night shifts. The disruption of circadian rhythms associated with such work schedules has been linked to a myriad of health complications, including immune dysregulation, which can compromise both the wellbeing of the nurses and quality of patient care (15). The economic implications of these health issues are profound, as they not only affect the individual nurses but also escalate healthcare costs due to increased absenteeism and diminished productivity (16). Despite the growing recognition of these challenges, current diagnostic and treatment strategies for immune-related disorders stemming from shift work remain inadequate, primarily addressing symptoms rather than the underlying molecular mechanisms (17–20).

In this study, we investigated the impact of shift work on the immune regulation of hospital nurses by analyzing the transcriptomic alterations in their peripheral blood mononuclear cells (PBMCs). Utilizing the GSE122541 dataset, we identified differentially expressed genes (DEGs) between day and night shift nurses, revealing significant associations with immune response pathways. Through Weighted Gene Co-expression Network Analysis (WGCNA), we discerned co-expression modules linked to night-shift work, ultimately identifying eight candidate hub genes, with *TRIM22* and *JAK1* emerging as pivotal regulators. Our findings underscore the intricate relationship between circadian rhythm disruption and changes in immune-related pathways, highlighting the necessity for further exploration of the underlying molecular mechanisms. The subsequent validation of these results in a murine model reinforces the relevance of *TRIM22* and *JAK1* in mediating the immune response alterations observed in night shift nurses, setting the stage for a deeper discussion on the implications of these findings for occupational health and potential therapeutic interventions.

The methodological strengths of this study lie in its comprehensive approach that integrates bioinformatics with experimental validation, thereby enhancing the robustness of the findings. By utilizing the GSE122541 dataset, we were able to identify DEGs in the PBMCs of hospital nurses engaged in night shifts, which provided a solid foundation for subsequent analyses. Application of WGCNA allowed for the identification of co-expression modules, revealing intricate relationships between gene expression patterns and shift work status. This method not only facilitated the discovery of 8 candidate hub genes but also underscored the significance of *TRIM22* and *JAK1* as central regulators in immune-related pathways associated with circadian disruption. Furthermore, the corroboration of these findings through circadian rhythm experiments in a murine model adds a critical layer of validation, reinforcing the biological relevance of the identified genes and their potential roles in immune signaling pathways.

The circadian rhythm experiment conducted in a murine model corroborated our findings, demonstrating that the downregulation of *TRIM22* and *JAK1* is a consistent feature of circadian disruption. This reinforces notion that these genes may serve as markers for immune dysregulation in shift workers and highlights the necessity for further exploration of their functional roles in immune signaling pathways, particularly those related to interferon-γ and NF-κB. Collectively, our findings contribute to a deeper understanding of molecular mechanisms underlying immune dysfunction in shift workers and underscore the importance of addressing these disruptions to improve the health outcomes of nurses engaged in night-shift work.

TRIM22 and JAK1 collaborate in immune regulation through distinct molecular mechanisms. TRIM22, as an E3 ubiquitin ligase, mainly exerts an antiviral effect in innate immunity and finely regulates the interferon signaling and inflammatory response through ubiquitination modification (21–24); while JAK1, as a key tyrosine kinase, is the core hub of signaling pathways of various cytokines (interferon, interleukin), and regulates the development, differentiation, and function of immune cells by activating the JAK-STAT pathway (25, 26). They are closely associated in the interferon response: the expression of TRIM22 is induced by the JAK-STAT pathway (21), and at the same time, it may feedback regulate through modifying the STAT and other proteins in this pathway, thereby jointly constituting a multi-level immune regulatory network (27), playing an important role in maintaining immune homeostasis and resisting infections. The imbalance of their functions is closely related to autoimmune diseases and chronic viral infections. Furthermore, existing studies have shown that interference with the JAK signaling pathway can also disrupt the circadian rhythm. Luo et al. demonstrated that miR-2799 can regulate the JAK/STAT pathway, thereby driving the rhythm of rest and activity in fruit flies (28).

While our research provides significant insights into the impact of shift work on immune regulation in hospital nurses, several limitations warrant consideration. Firstly, the relatively small sample size of the GSE122541 dataset, particularly the number of night-shift nurses (*n* = 9), may limit the generalizability of our findings. A larger cohort would enhance the robustness of the identified differentially expressed genes and their associated pathways. Additionally, while we utilized a murine model to validate our findings, the physiological differences between humans and mice may affect the translational relevance of our results. Lastly, the focus on PBMCs, while informative, does not encompass the full spectrum of immune responses that may occur in other immune cell types or tissues affected by circadian disruption. Future research should aim to address these limitations by incorporating larger, more diverse populations and exploring the broader implications of shift work on immune function across various biological systems.

Furthermore, although the sample size of this study was limited (nine night shift nurses vs. eight day shift nurses), it may affect the statistical test power and the generalizability of the results. However, we believe that this preliminary finding still has certain biological significance. Firstly, we adopted robust analysis methods such as WGCNA that are suitable for small samples, and conducted cross-validation using multiple algorithms to reduce the risk of false positives. More importantly, we independently verified the downregulation trend of core genes (Trim22 and Jak1) in the mouse circadian rhythm disorder model. Therefore, we tend to believe that although the sample size is small, combined with animal experiments and multi-omics analysis, this study still provides preliminary and credible molecular evidence. Future studies should be promoted and verified in larger-scale, multi-center nurse cohorts, and further explore the possibility of these genes as intervention targets.

In the subsequent experiments, the candidate genes TRIM22 and JAK1 need to undergo *in vitro* knockdown/overexpression experiments in peripheral blood mononuclear cells to verify their regulatory functions on immune-related signals, and to conduct functional validation using their inhibitors in the mouse model with disrupted circadian rhythm. Additionally, longitudinal samples will be collected from the on-duty nurses who work in a circadian rhythm-based shift system, including more clinical data (such as weight, cortisol levels, etc.) to directly assess the temporal causal relationship (29, 30).

## 5 Conclusions

In conclusion, our study demonstrates shift work, particularly night shifts, induces significant disruptions in immune-related transcriptome of PBMCs in nurses, as validated through integrated bioinformatics and animal experiments. The important finding is the identification of widespread differential gene expression enriched in critical immune pathways like T-cell receptor signaling, with further network analysis pinpointing *TRIM22* and *JAK1* as potential central hub genes that are consistently downregulated in night-shift nurses. Importantly, this downregulation was replicated in a murine circadian rhythm disorder experiment, confirming it as a core feature of circadian disruption rather than an isolated phenomenon. Mechanistically, these genes are implicated in interferon-γ and NF-κB signalings, providing a molecular link between shift work, circadian rhythm disturbance, and compromised immune regulation, which may underlie the increased health risks observed in this population.

## Data Availability

The original contributions presented in the study are included in the article/Supplementary material, further inquiries can be directed to the corresponding authors.
