# Supplementary material for: Shift work disrupts Immune regulation of the transcriptome in hospital nurses
Source: Front Public Health. 2026 Sep 22;14:1859265. doi: 10.3389/fpubh.2026.1859265 (PMC13638527; doi:10.3389/fpubh.2026.1859265)
Supplement: Supplementary file 1 [file Supplementary_file_1.docx]

**Supplementary Figures**

**Supplementary Figure 1**

**Supplementary Figure 1** A flowchart of the study design.

**Supplementary Figure 2**

**Supplementary Figure 2 (A, B)** Functional enrichment analysis included the GO term enrichment results of differential genes.

**Supplementary Figure 3**

**Supplementary Figure 3** The correlation analysis of the modules (blue, brown and red) and nurses with day-night shift.

**Supplementary Method**

***GEO dataset***

**GSE****122541**: Nine female, night-shift nurses (aged 29.6 ± 11.4; mean ± SD) and eight female, day-shift nurses (aged 34.9 ± 9.4) were recruited from the Hospital Nursing Staff. Participants met inclusion criteria if they worked full time (> 26h/week) as a hospital shift work nurse, were at least 19 y or older, able to schedule three consecutive work shifts (beginning or ending at 7:00 or 8:00) followed by at least three days off, had a current schedule that was consistent for the last three weeks, and at least 1 year of night-shift experience (night-shift nurses only).

On Day 9, a total of 48 mL of whole blood was harvested across eight sequential intervals, spaced three hours apart from 9:00 a.m. until 6:00 a.m., via an 18-gauge indwelling catheter; each draw yielded two aliquots: 1 mL designated for plasma separation and 5 mL reserved for peripheral blood mononuclear cell (PBMC) isolation. To maintain catheter patency between collections, a continuous infusion of 0.45% saline was administered at 20 mL/h. Immediately following collection, anticoagulated the whole blood underwent density gradient centrifugation to swiftly segregate cellular components, after which PBMCs were promptly cryopreserved.

***Identification of the differentially expressed genes***

Gene expression profile files were generated through normalizing the data and correcting the expression value in batches using the "sva" package. The LIMMA package was utilized to identify DEGs between the night-shift nurse group and day-shift nurse group, and a volcano plot was produced to highlight differential expression of DEGs. To control for the false positives caused by multiple testing, we employed the Benjamini-Hochberg (BH) method to perform multiple comparison correction on p-values, and set the threshold for selecting significantly differentially expressed genes as adjusted p-values (adj.P.Val) < 0.05 and |log2 fold change| > 1.

T-test Assumption: limma assumes gene expression data, after appropriate standardization, approximately follow a normal distribution, and estimates the gene-specific variance through empirical Bayesian shrinkage, thereby maintaining robustness even with a small sample size. We checked the standardization of the data (the original data had been processed by the original researchers using RMA or similar methods), and confirmed that the expression values, after log2 transformation, approximately satisfied normality. Additionally, limma does not require all genes to have same variance; its variance estimation method has good tolerance for heteroscedasticity.

***Functional enrichment analysis***

In order to conduct additional functional enrichment analysis, we are currently extracting genes from modules of interest. An examination using the Gene Ontology (GO) was carried out so that distinguishing features of the organism’s biological composition could be uncovered.

***Gene-MANIA analysis***

Gene-MANIA imports interaction networks from public databases and predicts these related genes for a query gene set. It integrates with Cytoscape for network visualization, analysis and automation. Gene-MANIA will find these genes that may share functions with it based on their interactions with it, and analyze and predict the functions of those genes. Gene network involved in this study consists of eight genes: *ROGDI*, *GAA*, *PKN1*, ***TRIM22***, *KRT1*, *LAX1*, *ARPC1B* and ***JAK1***.

***WGCNA analysis and module identification***

The WGCNA method augments the analysis of gene set expression. The WGCNA R package is employed to construct and modularize distinct gene networks across various stages. At first, before constructing the network, we retained at least those genes whose expression levels were greater than 0 in at least half of the samples. Eventually, this data matrix contained a total of 44 samples (Blood sampling samples taken from these 17 nurses at different time points) and 23,382 genes. The Pearson's correlation matrices and average linkage method were both performed for all pair-wise Genes,Then, a weighted adjacency matrix was constructed using a power function A_mn= |C_mn|^β (C_mn = Pearson's correlation between Gene_m and Gene_n; A_mn = adjacency between Gene m and Gene n). β was a soft-thresholding parameter that could emphasize strong correlations between Genes and penalize weak correlations. After choosing the power of 6, the adjacency was transformed into a topological overlap matrix (TOM), which could measure the network connectivity of a Gene defined as the sum of its adjacency with all other Genes for network Gene ration,and the corresponding dissimilarity (1-TOM) was calculated. To classify Genes with similar expression profiles into Gene modules,average linkage hierarchical clustering was conducted according to the TOM-based dissimilarity measure with a minimum size (Gene group) of 30 for the Genes dendrogram.

A cluster analysis is conducted on these samples to identify significant outliers. An automated network system is utilized to establish co-expression networks. These modules undergo functional evaluation through hierarchical clustering and dynamic tree cutting. The Module Membership (MM) and Gene Significance (GS) are assessed to correlate modules with clinical attributes. Modules displaying the highest Pearson Module Membership correlation (MM) and a p-value of 0.05 are classified as central modules. An MM value surpassing 0.8, coupled with a GS exceeding 0.1, indicates that the module demonstrates strong connectivity and clinical relevance. Gene information related to the pertinent modules is provided to promote further investigation. Genes within the clinically significant gene module network with a GS value exceeding 0.2 and an MM value greater than 0.8 are categorized as hub genes. Genes identified as overlapping are selected as candidates for crucial roles. Venn R package is employed to generate significant gene diagrams.

***Bioinformatics analysis***

All bioinformatics analyses were conducted on the online website "SangerBox **^[1]^**", including correlation analysis tools, functional analysis and WGCNA analysis. <http://sangerbox.com/>

***Reversed light-dark schedule experiment***

Through using commercialized rotating devices, the inherent biological clock rhythms of mice can be detected in a long-term artificially dark environment. Place the mice in a testing cage equipped with a rotating wheel for more than one week to allow them to adapt. Provide them with normal water and food, as well as a 12:12 light-dark cycle. Then, switch to a completely dark environment and continuously monitor the mice's activity and rest conditions. The light intensity was supplemented to 200 lux. The cage box is equipped with an independent ventilation system, maintaining a constant temperature of (22 ± 2°C) and humidity of (50 - 60%).

The specific steps are as follows: 6 female mice of 8-12 weeks of age for the experimental group and the control group respectively, and place them in mouse cages equipped with wheels for feeding. Fill the cages with water and food.

**Group A** (Day-Shift): The lighting schedule is set as turning on the lights at 8:00 every morning and turning them off at 20:00 every evening, for a total of one week.

**Group B** (Night-Shift): The lighting schedule is set as turning on the lights at 20:00 every evening and turning them off at 8:00 every morning, for a total of one week.

Sampling schedule

| Group | Lighting start time | Sampling time | Corresponding clock time | Physiological state |
| --- | --- | --- | --- | --- |
| A | 8:00 | 22:00 | 22:00 (The second hour of the Dark Period) | Active period |
| B | 20:00 | 10:00 (The next day) | 10:00 (The second hour of the Dark Period) | Active period |

The setting of the light frequency is based on the experiment conducted by Brooks et al **^[2]^**.

***RNA isolation and Quantitative RT-PCR***

Isolated PBMCs samples of the Circadian rhythm mouse, flash frozen in Trizol (Invitrogen™) were thawed on ice and transferred to Polystyrene tubes. RNA was extracted according to the manufacturer’s protocol. Candidate gene expression analysis was performed by qRT-PCR, using methods described previously.

RNA concentration and purity were assessed using a NanoDrop 2000 spectrophotometer (Thermo Fisher Scientific, USA), with A260/A280 ratios between 1.8 and 2.0. One microgram of total RNA was reverse-transcribed into cDNA using the PrimeScript™ RT Reagent Kit with gDNA Eraser (Takara, Cat# RR047A) in a 20 μL reaction volume, following the manufacturer’s protocol: 42°C for 2 min (gDNA elimination), 37°C for 15 min (reverse transcription), and 85°C for 5 s (enzyme inactivation).

Quantitative real-time PCR (qRT-PCR) was performed using TB Green® Premix Ex Taq™ II (Tli RNaseH Plus) (Takara, Cat# RR820A) on a StepOnePlus™ Real-Time PCR System. Each 20 μL reaction contained 10 μL of 2× TB Green Premix, 0.4 μL each of forward and reverse primers (10 μM), 2 μL of cDNA template, and 7.2 μL of RNase-free water. The thermal cycling conditions were as follows: initial denaturation at 95°C for 30 s; followed by 35 cycles of 95°C for 5 s and 60°C for 30 s; and a final dissociation stage (melt curve analysis) from 60°C to 95°C to confirm amplification specificity.

*Trim22:*

F：5’-CAGCTGAAGAGTTCCGAGTG-3’; R：5’-GCTCTGGATGGTCTTGTCTG-3’

*Jak1:*

F：5’-CAGCTGAAGAGTTCCGAGTG-3’; R：5’-GCTCTGGATGGTCTTGTCTG-3’

Gapdh was selected as the reference gene because its expression was stable across all samples, as confirmed by preliminary stability analysis using the geNorm algorithm. The relative expression of target genes was calculated using the 2^(−ΔΔCt) method, expressed as fold change relative to the day-shift (control) group. Each sample was run in technical triplicates, and the mean Ct value was used for analysis. For each group (night-shift or day-shift), n = 6 biological replicates (one mouse per replicate) were included. The specificity of each qRT-PCR product was verified by single-peak melt curve analysis and agarose gel electrophoresis (2% gel) to confirm the expected amplicon size.

**References**

1. Weitao Shen, Ziguang Song, Xiao Zhong, et al. Sangerbox: A comprehensive, interaction-friendly clinical bioinformatics analysis platform. *iMeta* 2022;1: e36.

<https://doi.org/10.1002/imt2.36>

2. Brooks JF 2nd, Behrendt CL, Ruhn KA, et al. The microbiota coordinates diurnal rhythms in innate immunity with the circadian clock. *Cell*. 2021;184(16):4154-4167.e12.
